# Leveraging existing biological knowledge in the identification of candidate genes for facial dysmorphology

**DOI:** 10.1186/1471-2105-10-S2-S12

**Published:** 2009-02-05

**Authors:** Hannah J Tipney, Sonia M Leach, Weiguo Feng, Richard Spritz, Trevor Williams, Lawrence Hunter

**Affiliations:** 1 Computational Pharmacology Department, University of Colorado at Denver and Health Sciences Center, Aurora, CO, USA; 2 ESAT, Research Division SCD, Katholieke Universiteit Leuven, B-3001 Leuven, Belgium; 3 Department of Craniofacial Biology, University of Colorado at Denver and Health Sciences Center, Aurora, CO, USA; 4 Human Medical Genetics Program, University of Colorado at Denver and Health Sciences Center, Aurora, CO, USA

## Abstract

**Background:**

In response to the frequently overwhelming output of high-throughput microarray experiments, we propose a methodology to facilitate interpretation of biological data in the context of existing knowledge. Through the probabilistic integration of explicit and implicit data sources a functional interaction network can be constructed. Each edge connecting two proteins is weighted by a confidence value capturing the strength and reliability of support for that interaction given the combined data sources. The resulting network is examined in conjunction with expression data to identify groups of genes with significant temporal or tissue specific patterns. In contrast to unstructured gene lists, these networks often represent coherent functional groupings.

**Results:**

By linking from shared functional categorizations to primary biological resources we apply this method to craniofacial microarray data, generating biologically testable hypotheses and identifying candidate genes for craniofacial development.

**Conclusion:**

The novel methodology presented here illustrates how the effective integration of pre-existing biological knowledge and high-throughput experimental data drives biological discovery and hypothesis generation.

## Background

The increased use of high-throughput analysis methods, such as microarrays, in mainstream biological research has led to a shift from studying small groups of reasonably well-characterized variables to exploring a complicated mire of thousands of inter-related variables simultaneously [1]. These methods are powerful, but their outputs are complicated and difficult to interpret due to the sheer volume of data produced. Interpretation can be prohibitively time consuming in the absence of computational assistance.

The ultimate goal of any microarray experiment is to gain insight into the workings of cellular organisms by understanding the interactions of genes and proteins. For this to be accomplished, raw data must not only be converted into information, but this information must also be interpreted in context, to be transformed into timely biological discovery and knowledge [2]. Currently, the lack of a community-wide consensus on how best to integrate experimental data with information resources limits this knowledge acquisition [2]. The recent work of Saraiya *et al. *(2005) [1] highlighted a "critical need" for tools able to "connect numerical patterns to the underlying biological phenomena", as current techniques fail to adequately link microarray data to biological meaning, which limits researchers' biological insights [1].

One intuitive way to integrate biological knowledge and microarray data is through protein-interaction networks, where nodes represent proteins and edges symbolize relationships between proteins [3]. However, focusing solely on physical protein interactions, such network constructs neglect a wealth of knowledge currently distributed among hundreds of existing biological databases (over 1000 listed in this year's *Nucleic Acids Research *database issue alone [4]) that is directly applicable to proteins investigated via microarray experiments. Current protein network constructs typically focus on a small subset of this biological knowledge, producing incomplete and sparsely populated resources. This is a particular problem for higher eukaryotic organisms such as mice and humans, for which physical protein interaction data are limited.

In agreement with Lee *et al. *(2004) [5] and Leach *et al. *(2007) [3], we demonstrate by expanding the definition of 'interaction' to include functional information that a) there is enough publicly available biological information to produce biologically useful, well populated interaction networks for higher eukaryotic species, b) through the combination of expression data and functional information, it is possible to provide contextual insight into the network, and c) it is possible to effectively link to existing biological knowledge using current technology. Using a murine craniofacial developmental expression microarray dataset [6] and a recently published technique for weighting and integrating functional interaction information [3], we illustrate how the application of context sensitive methodology leverages the full force of current available biological knowledge, enabling the translation of complex high-throughput datasets into scientific insight and discovery.

## Methods

### Microarray expression data

A comprehensive murine craniofacial developmental expression dataset was used in this study [6]. Expression was analyzed through the microdissection of mandibular, maxillary and frontonasal prominences at time points E10.5–E12.5 at 0.5 day increments. Expression was measured using the Affymetrix MOE430_2A microarray system. 916 microarray probes, corresponding to 712 unique MGI identifiers, were clustered using the MANOVA test statistic in R [7]. Hierarchical complete clustering was undertaken on the resulting correlation coefficients. The resultant tree was cut to produce 36 clusters.

### Explicit and implicit data sources

Traditionally an 'interaction' between two proteins is defined as a physical association. Here we expand the term to include functional relationships between pairs of proteins (encompassing any type of evidence, including physical, functional, genetic, biochemical, evolutionary, and computational evidence [3]). Functional interaction information was retrieved from a number of different resources falling into either of two categories: explicit and implicit. Explicit sources indicate a direct interaction between a pair of genes/proteins, and include experimentally measured physical, biochemical and genetic interactions, and computationally predicted gene neighborhoods, gene fusion events, or conserved phylogenetic profiles. Implicit sources provide information pertaining to an individual gene or protein attribute, which may be shared by any given pair of genes or proteins. Such attributes include literature references [8], sequence motifs (PReMod, InterPro) [9,10], protein categories (ChEBI) [11], protein complexes [12], phenotypes (as described by the Mouse Genome Database) [13], cellular location, molecular function, and biological processes (Gene Ontology) [14] and pathways (KEGG, Ingenuity) [3,15,16].

### Network construction, weighting and visualization

Genes within any given cluster were defined to be the nodes in our network constructs; a network was produced for each cluster identified from the hierarchical complete clustering stage. Data from both implicit and explicit sources were used to define arc interactions between pairs of proteins. Applying the cons NoisyOR methodology of Leach *et al. *(2007) [3], the edges between each pair of nodes were assigned a combined reliability score (network component, σ^*NET*^) based on the individual reliabilities of the sources asserting the edge [3]. Resultant networks were viewed using Cytoscape [17].

### Network identification and interrogation

**Figure 1:**
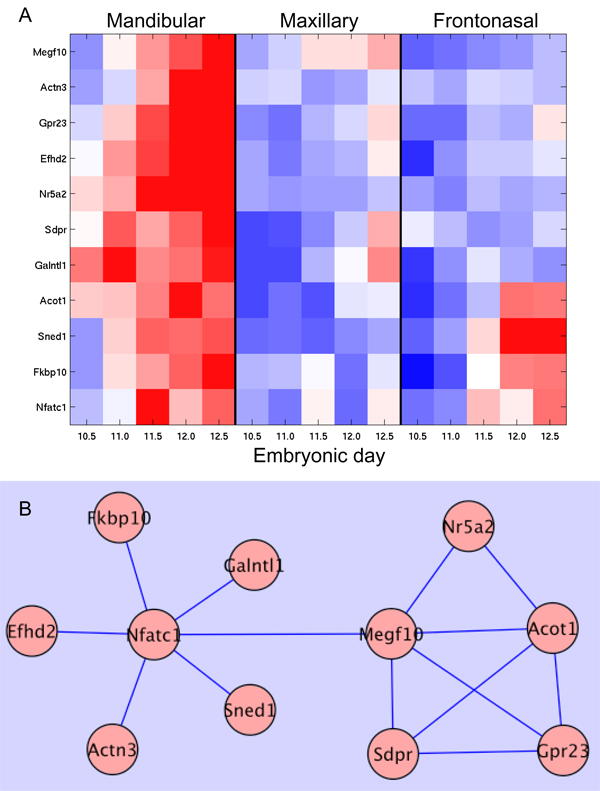
**ChEBI informed network**. A) Illustratates the mandibular-specific expression profile, B) the network of 11 nodes and their associated edges.

Based on significant tissue-restricted expression (expression limited to the mandibular prominence) and progressive increase in expression from embryonic day (E) 11 to E12.5 (Figure 1a), a network of interest was identified comprising 52 nodes.

By using Cytoscape's ability to display edge attributes, rapid orientation within the sub-network was achieved. Recognizing implicit functional themes common to nodes and edges across the sub-network facilitated the identification of key information such as shared pathways, processes, locations and phenotypes, as well as over-represented protein families. This approach provides a high-level overview of the networks functional composition, which subsequently directs the user towards more focused analysis of individual nodes and their pairwise interactions.

Each pair of nodes and associated interactions were reviewed through the exploration of 'links' within Cytoscape to a number of biological resources (including EntrezGene, GenBank, OMIM, PubMed, MGI, iHOP and UniGene). Although we were working with murine developmental expression microarray data, our goal was to gain insight into human developmental processes. Pertinent information is distributed across both mouse and human resources, thus requiring all investigations be repeated to ensure that valuable data were not overlooked simply due to their residing in a species-specific entry or database. In addition to mining these associated database entries, a corpus of relevant literature was compiled by following links from each implicit source. This corpus was then manually assessed and interpreted in order to extract key information.

The network construction method applied here provides a maximum of 12 possible functional interactions per edge. The edge with the most support in this network was between *Myod1 *[Myogenic differentiation 1; EntrezGene ID: 17927 (mouse) and 4654 (human)] and *Myog *[Myogenin; EntrezGene ID: 17928 (mouse) and 4656 (human)], and was asserted by nine sources (PubMed, PReMod, GO [BP, MF and CC], MGI Phenotype, InterPro, ZTransloc and ChEBI). Therefore, even with a structured interrogation strategy guided by both expression data and functional interactions, pursuing all informative leads was not a minor task. Considerable time was dedicated to the interpretation of the significance of each element. In this example, approximately 80 hours (10 days) of expert user time was required. The process is cyclical, where new information not only informs future discoveries, but previous work is frequently revisited to be viewed within new contexts.

## Results and discussion

### A mandibular-specific network: finding novelty

The group of 52 proteins was clustered on the basis of correlated mRNA expression, and with the construction of the network the goal was to develop biological hypotheses explaining the observed correlation. Those edges with the most support (in addition to correlated expression) frequently comprise well-documented relationships between the proteins encoded by these genes. Those edges with less support (fewer sources of shared characteristics) are therefore more likely to represent novel, as-yet uncharacterized relationships and so generate new hypotheses.

In this instance, a number of nodes (eleven) were linked by edges asserted by a single expert (ChEBI; Chemical Entities of Biological Interest). ChEBI is a dictionary of molecular entities focusing on 'small' chemical compounds of biological relevance and encompasses ontological classifications [11]. Interestingly, these nodes and edges also formed a discrete sub-network (Figure 1b). Although each of these nodes has reasonable amounts of associated biological knowledge, only the ChEBI data provided the shared functional interaction categorizations required for network construction (Table 1). This sub-graph of 11 nodes (linked by edges solely asserted by shared ChEBI categorizations), exhibiting correlated and progressively up-regulated expression in mandibular tissue during mouse development, thus provided a unique opportunity to search for truly novel biological hypotheses.

**Table 1:** Biological knowledge associated with each node.

ID	ChEBI	GO:BP	GO:CC	GO:MF	KEGG	PHENO
*Acot1*	✓	✓	✓	✓	-	-
*Actn3*	✓	✓	✓	✓	✓	-
*Efhd2*	✓	-	-	✓	-	-
*Fkbp10*	✓	✓	✓	✓	-	-
*Galntl1*	✓	-	✓	✓	✓	-
*Gpr23*	✓	✓	✓	✓	✓	-
*Megf10*	✓	✓	-	✓	-	-
*Nfatc1*	✓	✓	✓	✓	✓	-
*Nr5a2*	✓	✓	✓	✓	✓	✓
*Sdpr*	✓	-	✓	✓	-	-
*Sned1*	✓	✓	✓	✓	-	-

Data present represented by a ✓, absent by -.

### The common theme: calcium and lipids

By observing the sub-graph as a group of 11 interacting proteins (as opposed to individual or pairs of proteins), the implicit functional information displayed through the presence of edges can be taken together to produce a strong and unified voice capable of highlighting themes which may have otherwise gone unnoticed if only single, unassociated implicit resources were used.

In this instance, the ChEBI terms on which the network was constructed were "calcium(2+)" and "lipids". Cumulative evidence from GO and KEGG highlighted themes around muscle, acyl-CoA, lipids, signaling, and calcium signaling. Of the 11 nodes, only *Nr5a2 *[nuclear receptor subfamily 5, group A, member 2; EntrezGene ID: 26424 (mouse) and 2494 (human)] had a MGI phenotype association. *Nr5a2 *knockouts exhibit digestive, alimentary, and immune disruption, and are embryonic lethals.

### Insights from primary literature

Primary databases also provided a source of literature. Publications attached to GO annotations, GeneRIFs, and phenotypes (for example) can subsequently be explored further. Published literature is the gold standard for classification and description of biological functions; however, much of the knowledge in this vast resource is difficult to assess in the absence of prior knowledge of what to query for. Searching for pertinent information regarding all 52 associated genes in the original network and craniofacial disorder constitutes a formidable challenge due to the extremely large number of primary papers returned. However, the task becomes more manageable when the user has an insight into the relationships among a smaller subgroup of genes. In this instance, it was more productive to search over the ChEBI-specific sub-network for *Nfatc1*, a role in mandibular development, known associations with *Actn3*, *Sned1*, *Fkbp10*, *Galntl1*, *Efhd2*, and *Megf10*, and any associations with calcium signaling. By having a structured network rather than a gene list, mining the associated literature became a more targeted and thus more fruitful task.

**Figure 2:**
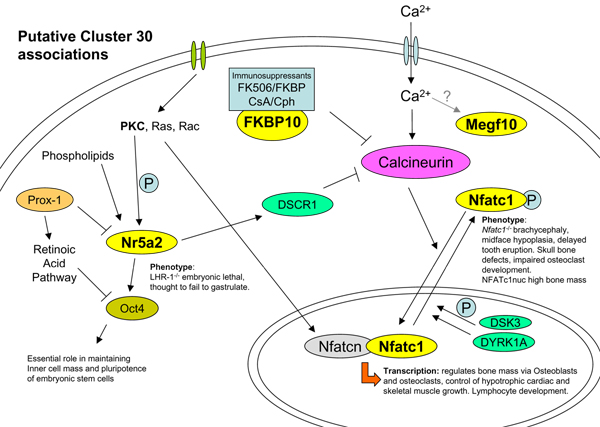
**Putative functional associations of ChEBI identified proteins**. From insights in the literature associations were found between Nfatc1, its phosphorylation status [19], calcineurin [20,21] and skeletal/craniofacial dysmorphology [19,22]; *Fkbp10*, its role in developing tissues [23] and negative regulation of calcineurin [20]; *Nr5a2*, its role in PKC-phosphorylation [24], DSCR1 expression [25](both PKC and DSCR1 are implicated in Nfatc-regulated transcription pathways [19,20]), and retinoic acid signalling [24,26](implicated in craniofacial development); α-actin (*Actn3*), its role in mediating calsarcin and calcineurin interactions [27]; and *Mef2*, its role in calcineurin-dependent gene-regulation [28]. *Megf10 *has putative a calcium binding site (IPR001881), while *Sned1 *and *Galntl1 *are found at the sites of embryonic apoptosis and ossification [29,30] but little more was discovered about these poorly characterised proteins. Yellow ovals highlight those proteins in the ChEBI sub-network.

As expected, the published literature provided a wealth of existing knowledge, and a putative biological pathway loosely centered on calcineurin was hypothesized to explain the correlated expression of the 11 genes (Figure 2). In the absence of a network to guide investigations, it would have been difficult, if not impossible, to associate these genes in a biologically meaningful way without domain-specific prior knowledge.

### The importance of leveraging pre-existing biological knowledge

Although the availability and application of high-throughput methods such as expression microarray technology has been a key advance in biological research, the biological research community is still grappling with how best to harness the power of this technology. Biologists are dependent upon computational methodologies to help them navigate and interpret their raw data, and as such the ability of the biologist to generate new insights, hypotheses and discoveries is intimately associated to the methodology's ability to assist effective discovery of biologically meaningful information. Saraiya *et al. *(2005) [1] highlighted how failing to link microarray data to existing biological knowledge prevents biologists from leveraging their domain expertise to construct higher level, biologically relevant hypotheses. They argued that it was "imperative that users be able to access and link biological information to their data" [1]. In agreement with this point, the study presented here illustrates how effective access to pre-existing knowledge can drive biological discovery. Inadequate access to this wealth of information ultimately hinders scientific discovery. We have demonstrated that through the combination of both explicit and implicit data and a permissive visualization environment such as Cytoscape, it is possible to link large-scale microarray datasets to biological information in a manner which facilitates hypothesis generation.

## Conclusion

The approach outlined here will be particularly useful when applied to analyses of large-scale datasets (such as from microarrays) to help understand the processes implicated in complex, multi-factorial disorders. In addition to the example presented here, application of this methodology to analysis of our craniofacial developmental expression microarray dataset [6] has led to identification and validation of four genes not previously implicated in craniofacial development [18]. We believe this methodology will be of significant use to the wider scientific community, and we are therefore also currently working towards explicitly capturing and automating this analysis protocol and developing a user interface to facilitate ease of investigation.

## Competing interests

The authors declare that they have no competing interests.

## Authors' contributions

HJT participated in the design of the system, undertook the analysis, and wrote the manuscript. SML designed and implemented the system. WF, TW and RS advised on and evaluated the biology, and critically appraised the manuscript. LH conceived of the system, supervised all aspects of its construction, and contributed to the manuscript.
